# Precise Method of Ambiguity Initialization for Short Baselines with L1-L5 or E5-E5a GPS/GALILEO Data

**DOI:** 10.3390/s20154318

**Published:** 2020-08-02

**Authors:** Mieczysław Bakuła

**Affiliations:** 1 Faculty of Geoengineering, University of Warmia and Mazury, 10-719 Olsztyn, Poland; mieczyslaw.bakula@uwm.edu.pl; 2 Institute of Navigation, Military University of Aviation, 08-521 Dęblin, Poland

**Keywords:** ambiguity function, L1-L5, E1-E5a, GPS, GALILEO, GNSS, PREFMAR

## Abstract

This paper presents a precise and fast method of ambiguity resolution (PREFMAR) for frequencies L1/E1 and L5/E5a of GPS/GALILEO data. The developed method is designed for precise and fast determination of ambiguities in GNSS phase observations. Ambiguities are chosen based on mathematical search functions. The fact that no variance–covariance matrix (VC matrix) with a so-called float solution is needed proves the innovativeness of the developed method. The developed method enables determination of the ambiguities for short baseline double-difference (DD) observations. The presented algorithms for the developed method enable unique and reliable calculation of the ambiguity if the actual errors of code measurements of DD observations are less than 0.38 m and the relative errors of phase observations are in the range of ±3 cm. The paper presents both mathematical derivations of the functions used in the PREFMAR and numerical calculations based on real double-difference GPS observations (L1-L5). The elaborated algorithms can be easily implemented into GNSS receivers or mobile phones. Therefore, they can be widely used in many geoscience applications, as well as in precise GPS/GALILEO navigation.

## 1. Introduction

GNSS positioning, with centimeter precision, using precise phase observations requires the inclusion of some unknown integer values, the so-called ambiguities, in satellite observations. Double-difference observations using two observation points and two satellites are used in relative positioning. For such an observation system, certain unknown N-values must be determined. These are constant over time if the observations are continuous and have no data gaps. In addition, if two frequencies are used, e.g., L1-L5, ambiguity needs to be determined for each frequency, i.e., $N_{L1}$ and $N_{L5}$. The problem of determining integer ambiguities of phase measurements in GPS observations dates back to 1978 [1,2]. Ambiguities are different for each pair of satellites and for each frequency, which poses an extremely complex mathematical and physical problem. Many scientists have worked to find the best and the most effective solution to this problem since the development of GPS systems [3]. In general, when using one or two frequencies, existing calculation methods can be divided into three groups:

- The first group of methods is based on linear combinations;
- The second group of methods uses the original search functions in 3D coordinate area;
- The third group is the most popular and employs the least-squares method.

The greatest step in ambiguity resolution was taken by Hatch–Melbourne–Wubbena [4,5,6] linear combinations, especially by using the so-called wide lane and narrow lane combinations. However, the first approach of ambiguity resolution was based on the use of mathematical functions [7]. Initially, the use of mathematical functions made it possible to achieve centimeter accuracy for vectors with a length of up to 10 km during static sessions lasting 2–3 h [8]. Later studies on mathematical functions improved efficiency of this approach [9,10,11]. The second method only uses the fractional part of the carrier phase measurements to minimize the objective function with the use of the least-squares estimation [12]. The third group is based on the integer least-squares estimation [13,14,15]. Methods of the first and the third group can effectively complement each other, especially in iterative computational strategies based on triple frequencies [16,17,18], in short baseline single-epoch solutions [19,20], and in longer baseline solutions [21]. 

Most ambiguity solutions start with the determination of estimated coordinates in the global XYZ system in order to determine the approximate ambiguity values, followed by a second and a third stage: estimation and validation. The second stage (estimation) is specific to various methods, as it indicates the most probable sets of ambiguities and is different in each of the ambiguity determination methods. The developed precise and fast method of ambiguity resolution (PREFMAR) uses mathematical functions to choose sets of ambiguities for GNSS observations transmitted on at least two frequencies. Therefore, each frequency combination has its own special properties and different efficiency in the reliable determination of unknown N-values. This research presents a method for determining the most probable ambiguities for GNSS signals transmitted on two frequencies: L1/E1 = 1575.42 MHz and L5/E5a = 1176.45 MHz. Signals of such frequencies are transmitted by GPS satellites, designated as L1 and L5, and GALILEO satellites, designated as E1 and E5a. Therefore, the L1/E1-L5/E5a frequency combination allows the integration of GPS and GALILEO for precise and reliable GNSS positioning [22,23,24].The first part of this contribution presents the mathematical background of the new method of ambiguity estimation based on new functions: $\Psi {\left(N_{L1}\right)}_{N_{L1}N_{L5}}$ and $\Psi {\left(N_{L5}\right)}_{N_{L5}N_{L1}}$. The second part of the work provides a detailed numerical example of ambiguity estimation ($N_{L1},N_{L5}$) based on real double-difference L1-L5 GPS data.

## 2. Observation of Double-Difference Equations for GPS Observations on L1 and L5 Frequencies and Their Intercorrelation

For GPS observations transmitted on two frequencies (L1 and L5), we can write the following observation equations for double-difference phase observations ($\phi_{L1},\phi_{L5}$) and code observations ($P_{L1},{\;P}_{L5}$) for a given measurement epoch (t):

(1) $$\lambda_{L1}\phi_{L1}\left(t\right)=\varrho \left(t\right)+\lambda_{L1}N_{L1}+\lambda_{L1}\varepsilon_{\phi_{L1}}\left(t\right)$$

(2) $$P_{L1}\left(t\right)=\varrho \left(t\right)+\varepsilon_{P_{L1}}\left(t\right)$$

(3) $$\lambda_{L5}\phi_{L5}\left(t\right)=\varrho \left(t\right)+\lambda_{L5}N_{L5}+\lambda_{L5}\varepsilon_{\phi_{L5}}\left(t\right)$$

(4) $$P_{L5}\left(t\right)=\varrho \left(t\right)+\varepsilon_{P_{L5}}\left(t\right)$$

where $\phi_{L1},\phi_{L5}$ are observations of double-difference (DD) phase measurements for L1 and L5 frequencies (in cycles), $P_{L1},P_{L5}$ are observations of DD in code measurements (m), $\varepsilon_{\phi_{L1}}$,$\varepsilon_{\phi_{L5}}\;$ are errors of phase DD observations, $\lambda_{L1}=$ 0.190293672798365 (m), and $\lambda_{L5}=0.254828048790854\;$(m).

There are three unknowns in the above equations: the value of ϱ(t) and the sought integral values $N_{L1}$ and $N_{L5}$. By separately examining the frequencies L1 and L5 (1-2) and (3-4), we can calculate the ambiguities $N_{L1}$ and $N_{L5}$ for a single measurement epoch, which are known in the literature as the so-called geometry-free (GF) integer solutions [25]:

(5) $$N_{L1}={\left[\phi_{L1}\left(t\right)-\frac{P_{L1}\left(t\right)}{\lambda_{L1}}\right]}_{\mathrm{roundoff}}$$

(6) $$N_{L5}={\left[\phi_{L5}\left(t\right)-\frac{P_{L5}\left(t\right)}{\lambda_{L5}}\right]}_{\mathrm{roundoff}}$$

Due to the transmission of signals at two frequencies at the same time, code and phase observations are strongly correlated. Similarly, as in Equation (6), Equation (5) may only be dependent on code measurements at frequency L5, as follows:

(7) $$N_{L1}={\left[\phi_{L1}\left(t\right)-\frac{P_{L5}\left(t\right)}{\lambda_{L1}}\right]}_{\mathrm{roundoff}}$$

**Figure 1:**
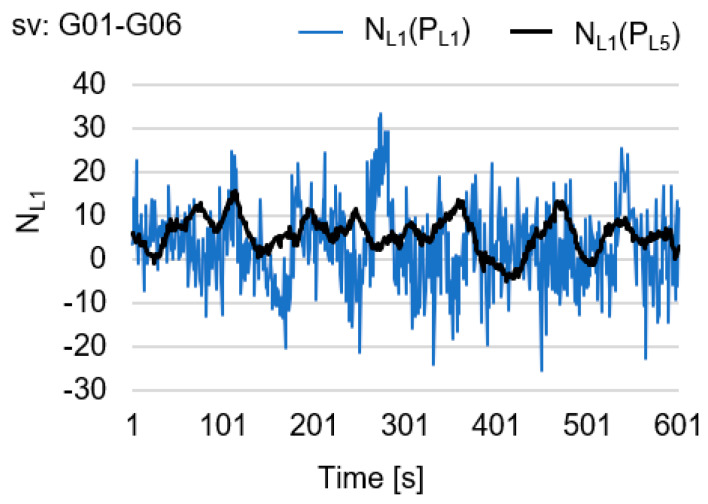
Time series of single-epoch double-difference (DD) geometry-free float solutions $N_{L1}$ with the use of $P_{L1}$ and $P_{L5}$.

Instead of value $P_{L5}\left(t\right)$, $P_{L1}\left(t\right)$ could be used, but practice shows that code observations ${\;P}_{L5}$ are much more accurate than $P_{L1}$ observations (see Figure 1). Code observations $P_{L5}$ are also much more accurate than $P_{L1}$ in smartphones [26]. The values of GF ambiguity for $N_{L1}$ using code $P_{L1}$ (based on Equation (5)) and $P_{L5}$ (based on Equation (7)) have been presented in Figure 1. The time series of single-epoch DD geometry-free float solutions presented in Figure 1 have been calculated using real GPS observations, made with Javad GNSS receivers, for a 10-min session with a measurement interval of 1 s.

.

**Figure 2:**
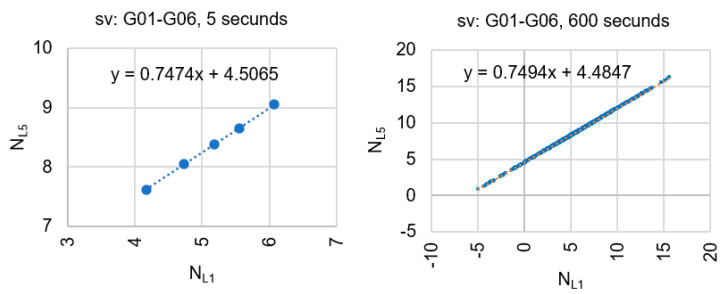
Scatter plot of correlated single-epoch geometry-free ambiguities $N_{L1}$ and $N_{L5}$.

GPS observations, expressed in formulas (6) and (7), are strongly correlated as they depend on the same code observation [27], e.g., observation $P_{L5}$. According to Teunissen’s research, strong correlation can also be obtained when the code $P_{L5}$ is replaced by a code $P_{L1}$ or by $2^{-1}\left(P_{L1}+P_{L5}\right)$. An example of correlated ambiguities for a pair of satellites (G01-G06) and observation $\phi_{L1},\phi_{L5},P_{L5}$ is shown in Figure 2, both for sessions with a length of five measurement epochs and for sessions of 600 observation epochs.

Figure 2 shows correlated ambiguities $N_{L1}$ and $N_{L5}$, using only the $P_{L5}$ code. Please note that the float ambiguities are arranged precisely along a straight line, both for 5-s and 10-min sessions. 

## 3. Ambiguity Regression Line Equations

If the correlated float ambiguities are arranged along a straight line, the regression equation for the ambiguity $N_{L5}$ in relation to ambiguity $N_{L1}$ in the ambiguity system $N_{L1}N_{L5}$ may be written as

(8) $$N_{L5}=a_{L1,L5}N_{L1}+b_{L1,L5}$$

or

(9) $${\tilde{N}}_{L5}=a_{L1,L5}{\tilde{N}}_{L1}+b_{L1,L5}$$

where

(10) $$a_{L1,L5}=\frac{f_{L5}}{f_{L1}}=\frac{\lambda_{L1}}{\lambda_{L5}}=\frac{115}{154}$$

As ambiguities in the form of real numbers (${\tilde{N}}_{L1},{\tilde{N}}_{L5}$) are located along a straight line expressed by (9), the value of $b_{L1,L5}$ can be calculated based on approximate float values (${\tilde{N}}_{L1,0},{\tilde{N}}_{L5,0}$):

(11) $$b_{L1,L5}={\tilde{N}}_{L5,0}-\frac{115}{154}{\tilde{N}}_{L1,0}$$

Thus, taking (11) into account, Equation (8) can be written as follows:

(12) $$N_{L5}=\frac{115}{154}(N_{L1}-{\tilde{N}}_{L1,0})+{\tilde{N}}_{L5,0}$$

If both integer $(N_{L5}$) and float $({\tilde{N}}_{L5}$) ambiguities lie on the same straight line, the ${\tilde{N}}_{L5}$ can also be written as

(13) $${\tilde{N}}_{L5}=\frac{115}{154}(N_{L1}-{\tilde{N}}_{L1,0})+{\tilde{N}}_{L5,0}$$

Thus, Equations (12) and (13) represent regression line equations for both real ambiguities (13) and the integer ambiguities (12) and are dependent on approximate values of ${\tilde{N}}_{L1,0}$ and ${\tilde{N}}_{L5,0}$.

## 4. Ambiguity Functions for L1-L5 GPS Data

Let us, therefore, define a certain function, $\Psi {\left(N_{L1}\right)}_{N_{L1}N_{L5}}$, as follows:

(14) $$\Psi {\left(N_{L1}\right)}_{N_{L1}N_{L5}}=\lambda_{L5}\left({\tilde{N}}_{L5}-{\left[{\tilde{N}}_{L5}\right]}_{\mathrm{roundoff}}\right)$$

Equation (14), with Equation (13) taken into account, for the system $N_{L1}N_{L5}\;$ can be written as follows:

(15) $$\Psi {\left(N_{L1}\right)}_{N_{L1}N_{L5}}=\lambda_{L5}\left({\tilde{N}}_{L5}-{\left[{\tilde{N}}_{L5}\right]}_{\mathrm{roundoff}}\right)\;=\lambda_{L5}\left(\frac{115}{154}(N_{L1}-{\tilde{N}}_{L1,0})+{\tilde{N}}_{L5,0}-{\left[\frac{115}{154}(N_{L1}-{\tilde{N}}_{L1,0})+{\tilde{N}}_{L5,0}\right]}_{\mathrm{roundoff}}\right)$$

where ${\tilde{N}}_{L1,0}$ and ${\tilde{N}}_{L5,0}$ represent a float solution or are calculated as follows:

(16) $${\tilde{N}}_{L1,0}=\phi_{L1}\left(t\right)-\frac{P_{L5}\left(t\right)}{\lambda_{L1}}$$

(17) $${\tilde{N}}_{L5,0}=\phi_{L5}\left(t\right)-\frac{P_{L5}\left(t\right)}{\lambda_{L5}}$$

Similarly, for system $N_{L5}N_{L1}$ and function $\Psi {\left(N_{L5}\right)}_{N_{L5}N_{L1}}$ we obtain

(18) $$\Psi {\left(N_{L5}\right)}_{N_{L5}N_{L1}}=\lambda_{L1}\left({\tilde{N}}_{L1}-{\left[{\tilde{N}}_{L1}\right]}_{\mathrm{roundoff}}\right)\;=\lambda_{L1}\left(\frac{154}{115}(N_{L5}-{\tilde{N}}_{L5,0})+{\tilde{N}}_{L1,0}-{\left[\frac{154}{115}(N_{L5}-{\tilde{N}}_{L5,0})+{\tilde{N}}_{L1,0}\right]}_{\mathrm{roundoff}}\right)$$

**Figure 3:**
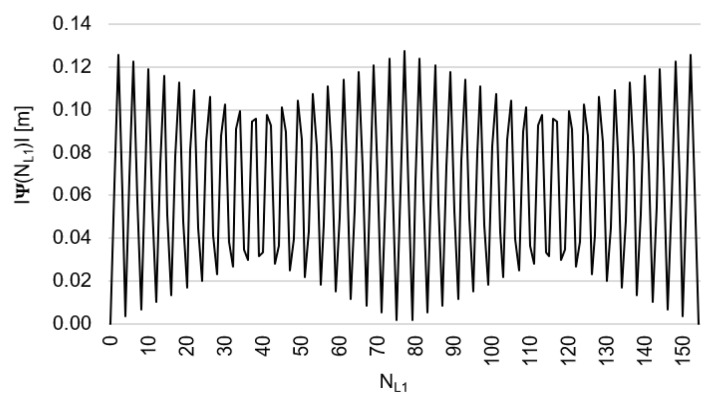
Behavior of a full period of the $\left|\Psi {\left(N_{L1}\right)}_{N_{L1}N_{L5}}\right|$ function in the $N_{L1}N_{L5}$ system, in the $N_{L1}\in <0;154>$ interval, for GNSS observations and frequencies L1/E1 and L5/E5a.

**Figure 4:**
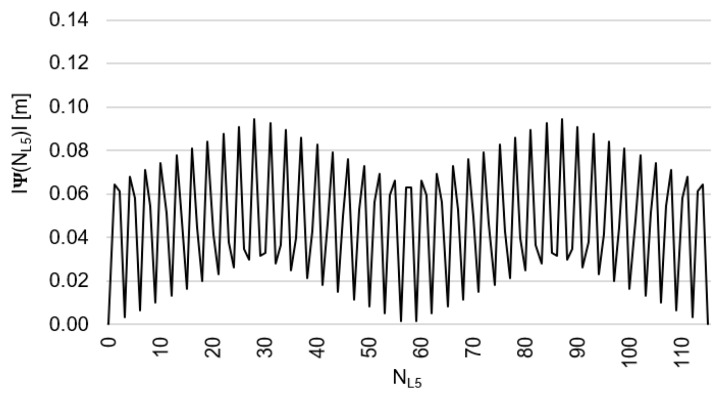
Behavior of a full period of the function $\left|\Psi {\left(N_{L5}\right)}_{N_{L5}N_{L1}}\right|$ in the $N_{L5}N_{L1}$ system, in the $N_{L5}\in <0;115>$ interval, for GNSS observations and frequencies L1/E1 and L5/E5a.

Behaviors of the functions $\left|\Psi {\left(N_{L1}\right)}_{N_{L1}N_{L5}}\right|$ and $\left|\Psi {\left(N_{L5}\right)}_{N_{L5}N_{L1}}\right|$ have been presented in Figure 3 and Figure 4, where their minima, and thus their periodic character (repeatability), can be clearly seen. The horizontal axes represent integer candidates, but the vertical ones represent values of the ambiguity functions.

Based on an analysis of values of the function $\Psi {\left(N_{L1}\right)}_{N_{L1}N_{L5}}$, in the (−0.127; +0.126 m), interval, it can be observed that the values of the function $\Psi {\left(N_{L1}\right)}_{N_{L1}N_{L5}}$ for $f_{L1}$ and $f_{L5}$ frequencies of GPS observations repeat precisely every 154 $N_{L1}$ cycles. Values of the function $\Psi {\left(N_{L5}\right)}_{N_{L5}N_{L1}}$ are in the interval (−0.094; +0.094 m) and repeat precisely every 115 $N_{L5}\;$ cycles. The functions $\Psi {\left(N_{L1}\right)}_{N_{L1}N_{L5}}$ and $\Psi {\left(N_{L5}\right)}_{N_{L5}N_{L1}}$ have the same wavelength ($\lambda_{\Psi }=$ 29.3052256109482 m) and frequency ($f_{\Psi }=$10.23 MHz) and are equivalent in the process of determining ambiguities $N_{L1}$ and $N_{L5}$, for values

(19) $$\left|\varepsilon_{L1,L5}\right|\leq 0.5\lambda_{L1}\leq 0.0951468363991824\;m$$

where $\varepsilon_{L1,L5}$ represents both relative errors in phase observations, i.e.,

(20) $$\varepsilon_{L1,L5}\left(t\right)=\lambda_{L5}\varepsilon_{\phi_{L5}}\left(t\right)-\lambda_{L1}\varepsilon_{\phi_{L1}}\left(t\right)$$

and the value $\varepsilon_{L1,L5}\;$ meets the relation expressed by Equation (14), i.e.,

(21) $$\varepsilon_{L1,L5}=\Psi {\left(N_{L1}\right)}_{N_{L1}N_{L5}}=\lambda_{L5}\left({\tilde{N}}_{L5}-{\left[{\tilde{N}}_{L5}\right]}_{\mathrm{roundoff}}\right)$$

Selection of the most likely sets of ambiguities ($N_{L1}^{i},N_{L5}^{i}$) in the PREFMAR depends on approximate values of the float solution (${\tilde{N}}_{L1,0},\;{\tilde{N}}_{L5,0}$) and relative error values in L1 and L2 carrier phase observations.

## 5. Ambiguity Search Space in the PREFMAR

Generally, we can assume that the sought integer ambiguities $N_{L1}$ and $N_{L5}$ lie exactly on the regression line, which additionally needs to pass through an unknown and sought point with coordinates ${\hat{N}}_{L1}\;$ and ${\hat{N}}_{L5}$. Unfortunately, this is only when (I) $\varepsilon_{\phi_{L1}}=0$ and $\varepsilon_{\phi_{L5}}=0,$ and therefore when the errors in phase observations are equal to zero, or (II) when the relative errors in phase observations are equal to zero, i.e., $\varepsilon_{L1,L5}=0$. The first situation corresponds to ideal phase observations, whereas the second one is in the situation when $\lambda_{L1}\varepsilon_{\phi_{L1}}=\lambda_{L5}\varepsilon_{\phi_{L5}}$. Detailed analysis of the functions $\left|\Psi {\left(N_{L1}\right)}_{N_{L1}N_{L5}}\right|$ and $\left|\Psi {\left(N_{L5}\right)}_{N_{L5}N_{L1}}\right|$ shows that the minima of the functions $\left|\Psi {\left(N_{L1}\right)}_{N_{L1}N_{L5}}\right|$ and $\left|\Psi {\left(N_{L5}\right)}_{N_{L5}N_{L1}}\right|$ indicate the sought ambiguity values $({\hat{N}}_{L1}\;$ and ${\hat{N}}_{L5}$), but only if the errors in phase observations are equal to or close to zero. However, the question that remains is how big the errors in DD phase observations can be for the ambiguities to be determined by the minima of these functions. In the case of phase measurements L1-L5, this depends on the smallest value of the function $\Psi {\left(N_{L1}\right)}_{N_{L1}N_{L5}}$ or $\Psi {\left(N_{L5}\right)}_{N_{L5}N_{L1}}$, and these are the values $\Delta_{\Psi {\left(N_{L1}\right)}_{N_{L1}N_{L5}}}$ and $\Delta_{\Psi {\left(N_{L5}\right)}_{N_{L5}N_{L1}}}$, which we calculate as

(22) $$\Delta_{\Psi {\left(N_{L1}\right)}_{N_{L1}N_{L5}}}=\Delta_{\Psi {\left(N_{L5}\right)}_{N_{L5}N_{L1}}}=\frac{1}{154}\lambda_{L5}=\frac{1}{115}\lambda_{L1}=0.0016\;m$$

The formula (22), therefore, defines the smallest unit of function $\Psi {\left(N_{L1}\right)}_{N_{L1}N_{L5}}$ in the $N_{L1}N_{L5}$ system and the smallest unit of function $\Psi {\left(N_{L5}\right)}_{N_{L5}N_{L1}}$ in the $N_{L5}N_{L1}$ system. Thus, if the relative errors in DD phase observations are less than half of this unit (22), i.e., less than 0.0008 m, then the minima of the functions $\left|\Psi {\left(N_{L1}\right)}_{N_{L1}N_{L5}}\right|$ and $\left|\Psi {\left(N_{L5}\right)}_{N_{L5}N_{L1}}\right|$ determine, on the horizontal axis, the sought ambiguities, with $N_{L1}$ using the minimum of the $\left|\Psi {\left(N_{L1}\right)}_{N_{L1}N_{L5}}\right|$ function and $N_{L5}$ using the minimum of the $\left|\Psi {\left(N_{L5}\right)}_{N_{L5}N_{L1}}\right|$ function. In both cases, approximate values ${\tilde{N}}_{L1,0}\;$ and ${\tilde{N}}_{L5,0}\;$ must be at a distance of less than 14.653 m from the true values sought (${\hat{N}}_{L1}$ and ${\hat{N}}_{L5}$). 

If, however, the absolute values $\Delta_{\Psi {\left(N_{L1}\right)}_{N_{L1}N_{L5}}}$ or $\Delta_{\Psi {\left(N_{L5}\right)}_{N_{L5}N_{L1}}}$ are different from zero and larger than 0.0008 m, then ambiguity values will be located at different points than the minima of the $\left|\Psi {\left(N_{L1}\right)}_{N_{L1}N_{L5}}\right|$ and $\left|\Psi {\left(N_{L5}\right)}_{N_{L5}N_{L1}}\right|$ functions, and their selection will strongly depend on the real values $\varepsilon_{L1,L5}$ and on the approximate ${\tilde{N}}_{L1,0}\;$ and ${\tilde{N}}_{L5,0}\;$ values.

When analyzing the graph of the function $\Psi {\left(N_{L1}\right)}_{N_{L1}N_{L5}}$ in the system $N_{L1}N_{L5}\;$ and the graph of the function $\;\Psi {\left(N_{L5}\right)}_{N_{L5}N_{L1}}$ in the system $N_{L5}N_{L1}$, we should start the search for the first pair of ambiguities [$N_{L1}^{I}$, $N_{L5}^{I}$] in the area of errors of code measurements below $2\lambda_{L1}$ float [${\tilde{N}}_{L1,0}$, ${\tilde{N}}_{L5,0}$], i.e., $\left|\left(N_{L1}^{I}-{\tilde{N}}_{L1,0}\right)\right|<2\lambda_{L1},\;$ and for relative errors of phase observations $\;\varepsilon_{L1,L5}\subset <-32\;mm;+32\;mm>$; we perform the search for the second pair [$N_{L1}^{II}$, $N_{L5}^{II}$] in the area $\;4\lambda_{L1}$, i.e., $\left|\left(N_{L1}^{II}-{\tilde{N}}_{L1,0}\right)\right|<4\lambda_{L1}$; while the third pair [$N_{L1}^{III}$, $N_{L5}^{III}$] of ambiguities must be at a distance of $6\lambda_{L1}$ from the value ${\tilde{N}}_{L1,0}$ and ${\tilde{N}}_{L5,0}$.

**Figure 5:**
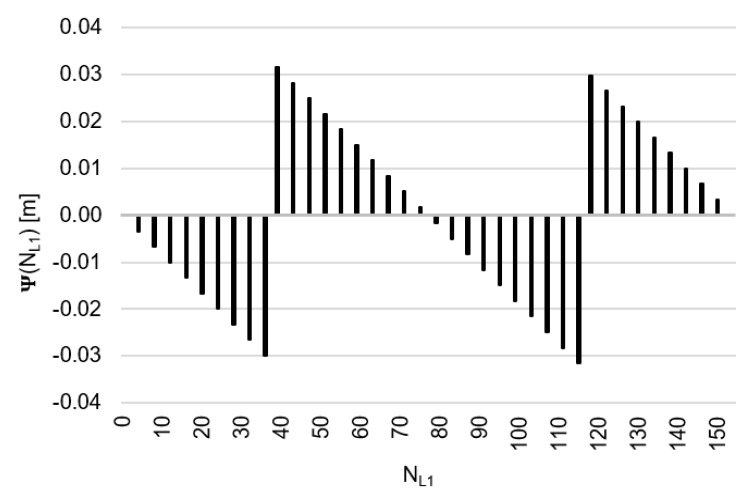
Behavior of the function $\Psi {\left(N_{L1}\right)}_{N_{L1}N_{L5}}$ (m), for values from interval <−32;+32 mm>.

**Figure 6:**
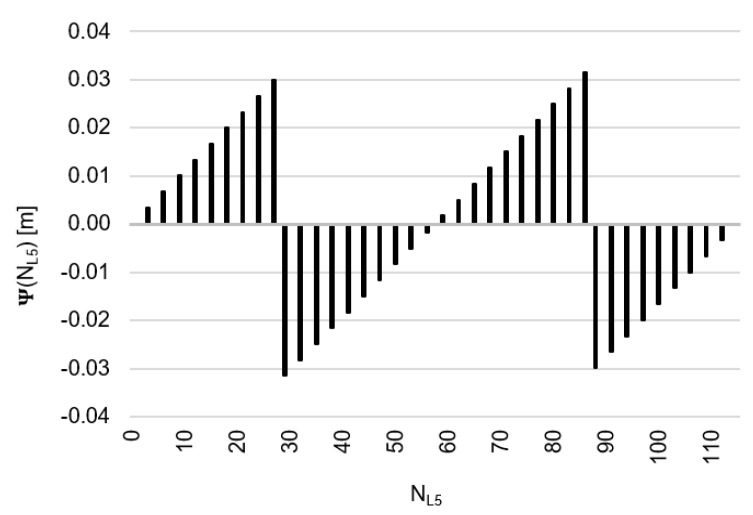
Behavior of the function $\Psi {\left(N_{L5}\right)}_{N_{L5}N_{L1}}$ (m), for values from the interval <−32;+32 mm>.

**Figure 7:**
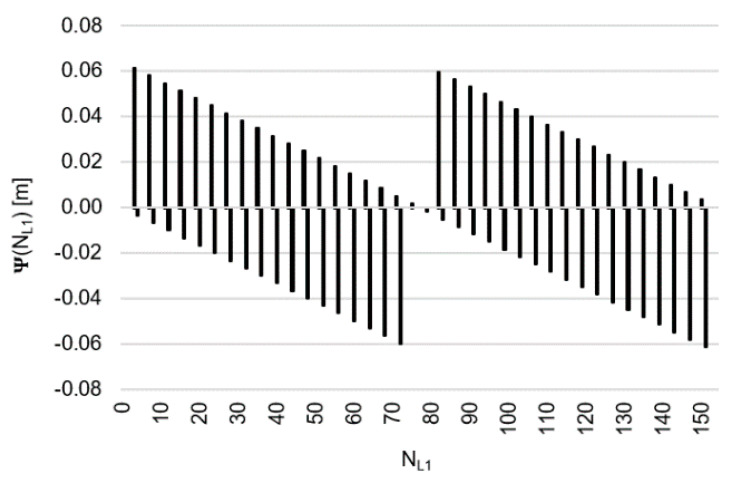
Behavior of the function $\Psi {\left(N_{L1}\right)}_{N_{L1}N_{L5}}$ (m), for values from interval <−62;+62 mm>.

**Figure 8:**
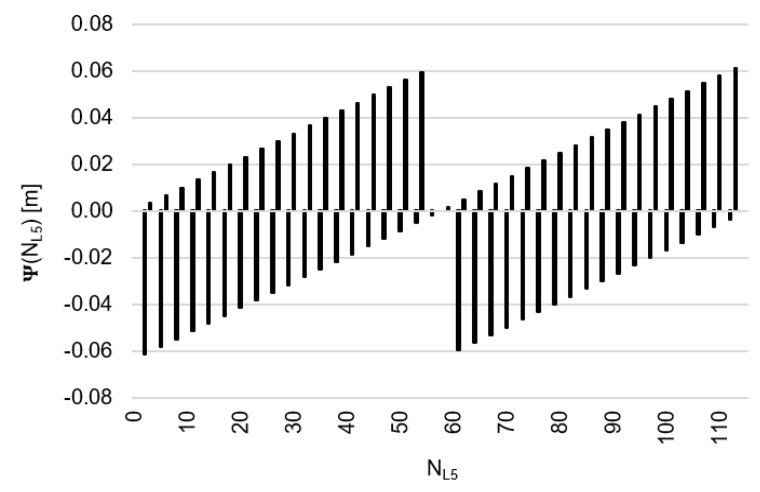
Behavior of the function $\Psi {\left(N_{L5}\right)}_{N_{L5}N_{L1}}$ (m), for values from interval <−62;+62 mm>.

Let us, therefore, assume that the values of the function $\Psi {\left(N_{L1}\right)}_{N_{L1}N_{L5}}$ lie within the range ±32 mm, then the function $\Psi {\left(N_{L1}\right)}_{N_{L1}N_{L5}}$ behaves as shown in Figure 5. Similarly, for the function $\;\Psi {\left(N_{L5}\right)}_{N_{L5}N_{L1}}$, we assume a range of values of ±32 mm (Figure 6). Please note that for relative errors in DD phase observations with values up to ±3 cm, the ambiguity $N_{L1}$ changes exactly by four cycles, whereas ambiguity $N_{L5}$ changes by three cycles. For comparison, the search areas for $\;\Psi {\left(N_{L1}\right)}_{N_{L1}N_{L5}}$ and $\Psi {\left(N_{L5}\right)}_{N_{L5}N_{L1}}$ which lie within ±62 mm have been presented in Figure 7 and Figure 8.

For relative errors of carrier phases (20) in the range <−32;+32 mm>, these will be the following proposals for $N_{L1}$ (Figure 5) and for $N_{L5}$ (Figure 6):

$$N_{L1}\in \left\{\begin{array}{c} 0;4;8;12;16;20;24;28;32;36;39;43;47;\\ 51;55;59;63;67;71;75;79;83;87;91;95;99;\;\\ 103;107;111;115;118;122;126;130;134;138;\\ 142;146;150;154 \end{array}\right\},\;N_{L5}\in \left\{\begin{array}{c} 0;3;6;9;12;15;18;21;24;27;29;32;35;\\ 38;41;44;47;50;53;56;59;62;65;68;71;74;\;\\ 77;80;83;86;88;91;94;97;100;103;\\ 106;109;112;115 \end{array}\right\}$$

Figure 7 and Figure 8 show a template of possible ambiguities for relative errors in DD phase observations up to ±62 mm, which seems to be sufficient from a practical point of view even for baselines of several kilometers or much more and for noisy GPS/GALILEO data in mobile phones.

## 6. Calculation Scheme for N Measurement Epochs Using the PREFMAR

For continuous GNSS observations, and using more than one measurement epoch, general formulas for the PREFMAR are used, which can be summarized in the following main points:

- Calculation of correlated approximate values of ${\tilde{N}}_{L1,0}$ and ${\tilde{N}}_{L5,0}$.
  Determination of float value based on the global solution ${\tilde{N}}_{L1,0}\;$ and ${\tilde{N}}_{L5,0}$, or using only DD observations for any pair of satellites (for n epochs), i.e.,
  (23) $${\tilde{N}}_{L1,0}=n^{-1}\overset{n}{\underset{i=1}{\sum }}\left(\phi_{L1,i}-\frac{P_{L5}}{\lambda_{L1}}\right)$$
  (24) $${\tilde{N}}_{L5,0}=n^{-1}\overset{n}{\underset{i=1}{\sum }}\left(\phi_{L5,i}-\frac{P_{L5}}{\lambda_{L5}}\right)$$
- Regression line estimation.
  (25) $${\tilde{N}}_{L5}=\frac{115}{154}(N_{L1}-{\tilde{N}}_{L1,0})+{\tilde{N}}_{L5,0}$$
  (26) $${\tilde{N}}_{L1}=\frac{154}{115}(N_{L5}-{\tilde{N}}_{L5,0})+{\tilde{N}}_{L1,0}$$
- Determination of search functions $\Psi {\left(N_{L1}\right)}_{N_{L1}N_{L5}}$ (m) or $\Psi {\left(N_{L5}\right)}_{N_{L5}N_{L1}}$ (m).
  (27) $$\Psi {\left(N_{L1}\right)}_{N_{L1}N_{L2}}==\lambda_{L5}\left(\frac{115}{154}(N_{L1}-{\tilde{N}}_{L1,0})+{\tilde{N}}_{L5,0}-{\left[\frac{115}{154}(N_{L1}-{\tilde{N}}_{L1,0})+{\tilde{N}}_{L5,0}\right]}_{\mathrm{roundoff}}\right)$$
  (28) $$\Psi {\left(N_{L5}\right)}_{N_{L5}N_{L1}}==\lambda_{L1}\left(\frac{154}{115}(N_{L5}-{\tilde{N}}_{L5,0})+{\tilde{N}}_{L1,0}-{\left[\frac{154}{115}(N_{L5}-{\tilde{N}}_{L5,0})+{\tilde{N}}_{L1,0}\right]}_{\mathrm{roundoff}}\right)$$
- Selection of the most likely sets of ambiguities, depending on approximate values (${\tilde{N}}_{L1,0}$,${\tilde{N}}_{L5,0}$) and using function $\;\Psi {\left(N_{L1}\right)}_{N_{L1}N_{L5}}$ or function $\Psi {\left(N_{L5}\right)}_{N_{L5}N_{L1}}$.

For short baselines, and assuming that $\varepsilon_{L1,L5}\subset <-32;+32\;\mathrm{mm}>$, the four most likely sets of ambiguities ($N_{L1}^{i},N_{L5}^{i}$) will be at a distance of up to 1.55 m from the float value and will be selected as follows:

(29) $$\mathrm{Solution}\;\mathrm{no}\;I:=\{\begin{array}{c} N_{L1}^{I}\\ N_{L5}^{I} \end{array},\;\mathrm{where}\;\left|\left(N_{L1}^{I}-{\tilde{N}}_{L1,0}\right)\right|<2\lambda_{L1},\;(<0.381\;m)$$

(30) $$\mathrm{Solution}\;\mathrm{no}\;\mathrm{II}:=\{\begin{array}{c} N_{L1}^{II}\\ N_{L5}^{II} \end{array},\;\mathrm{where}\;\left|\left(N_{L1}^{II}-{\tilde{N}}_{L1,0}\right)\right|<4\lambda_{L1},\;(<0.761\;m)$$

(31) $$\mathrm{Solution}\;\mathrm{no}\;\mathrm{III}:=\{\begin{array}{c} N_{L1}^{III}\\ N_{L5}^{III} \end{array},\;\mathrm{where}\;\left|\left(N_{L1}^{III}-{\tilde{N}}_{L1,0}\right)\right|<6\lambda_{L1},\;(<1.142\;m)$$

(32) $$\mathrm{Solution}\;\mathrm{no}\;\mathrm{IV}:=\{\begin{array}{c} N_{L1}^{IV}\\ N_{L5}^{IV} \end{array},\;\mathrm{where}\;\left|\left(N_{L1}^{IV}-{\tilde{N}}_{L1,0}\right)\right|<8\lambda_{L1},(<1.552\;m)$$

However, if we assume that the relative errors $\varepsilon_{L1,L5}\subset <-62;+62\;\mathrm{mm}>$, then solutions I and II remain the same as for $\varepsilon_{L1,L5}\subset <-32;+32\;\mathrm{mm}>$, whereas solutions III and IV are at a distance of 3$\lambda_{L1}$ and 4$\lambda_{L1}$ from the float value, respectively. Limiting the search range to 4$\lambda_{L1}$ requires an accuracy of the float position of about 0.76 m if the search range is set for $\varepsilon_{L1,L5}\subset <-62;+62\;\mathrm{mm}>$. However, given the number of satellites currently available, obtaining an accuracy of the float solution better than 0.76 m should not be a problem, which allows a quick indication of the most likely ambiguities for validation, even for GNSS measurements under difficult observational conditions or for mobile phones. However, based on preliminary tests, for high-quality GNSS receivers, the search area of ambiguity resolution $N_{L1}$ and $N_{L5}$ for relative errors $\varepsilon_{L1,L5}\subset <-32;+32\;\mathrm{mm}>$ should be sufficient for short baseline RTK positioning.

## 7. Numerical Example Using the PREFMAR and Float Solution

The formulas presented for the developed method were applied to real GPS data, for a very short baseline, based on DD observations for the G01–G06 satellite pair, using five observation epochs. The DD observations for these satellites are shown in Table 1.

Sets of the most probable ambiguities ($N_{L1}$,$N_{L5}$) can be determined both with the use of function $\Psi {\left(N_{L1}\right)}_{N_{L1}N_{L5}}$ and function $\Psi {\left(N_{L5}\right)}_{N_{L5}N_{L1}}$. Let us thus present the necessary calculations in the form of Table 2 for the function $\Psi {\left(N_{L1}\right)}_{N_{L1}N_{L5}}$ and using 5 DD observations for which the float values are respectively equal to ${\tilde{N}}_{L1,0}=5.141$ and ${\tilde{N}}_{L5,0}=8.349$. Then, for subsequent integer values $N_{L1}$, located around the float solution ${\tilde{N}}_{L1,0}$, within the range of, e.g., ±8 $N_{L1}$ cycles (i.e., at a distance of 1.5 m) from approximate values of ${\tilde{N}}_{L1,0}=$ 5.141, we calculate elements of the column ${\tilde{N}}_{L5,i}$ using the formula

(33) $${\tilde{N}}_{L5,i}=\frac{115}{154}*N_{L1,i}+8.349-\frac{115}{156}*5.141$$

and the values of the $\Psi {\left(N_{L1}\right)}_{N_{L1}N_{L5}}$ function are calculated with the formula

(34) $$\begin{array}{cc} \Psi {\left(N_{L1}\right)}_{N_{L1}N_{L5}}= & \lambda_{L5}(\frac{115}{154}N_{L1,i}+8.349-\frac{115}{154}*5.141\\ & -{\left[\frac{115}{154}N_{L1,i}+8.349-\frac{115}{154}*5.141\right]}_{\mathrm{roundoff}}) \end{array}$$

Similarly, for the function $\Psi {\left(N_{L5}\right)}_{N_{L5}N_{L1}}$, the calculations are shown in Table 3. The values of the function $\Psi {\left(N_{L5}\right)}_{N_{L5}N_{L1}}$ are calculated from the following formula:

(35) $$\begin{array}{cc} \Psi {\left(N_{L5}\right)}_{N_{L5}N_{L1}}= & \lambda_{L1}(\frac{154}{115}N_{L5}+5.141-\frac{154}{115}*8.349\\ & -{\left[\frac{154}{115}N_{L5}+5.141-\frac{154}{115}*8.349\right]}_{\mathrm{roundoff}}) \end{array}$$

**Figure 9:**
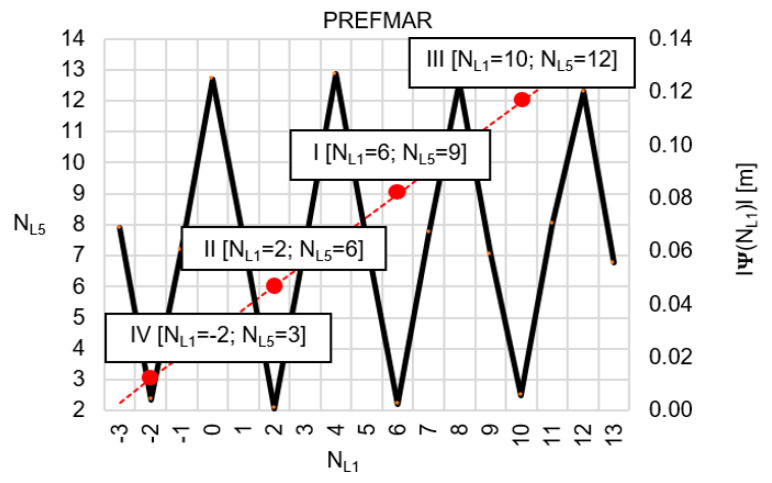
Search area of ambiguities in $N_{L1}N_{L5}$ system, for ${\tilde{N}}_{L1,0}=5.141$ and ${\tilde{N}}_{L5,0}=8.349$, with the use of the function $\left|\Psi {\left(N_{L1}\right)}_{N_{L1}N_{L5}}\right|$, for $\left|\varepsilon_{L1,L5}\right|<5.5\;\mathrm{cm}$.

**Figure 10:**
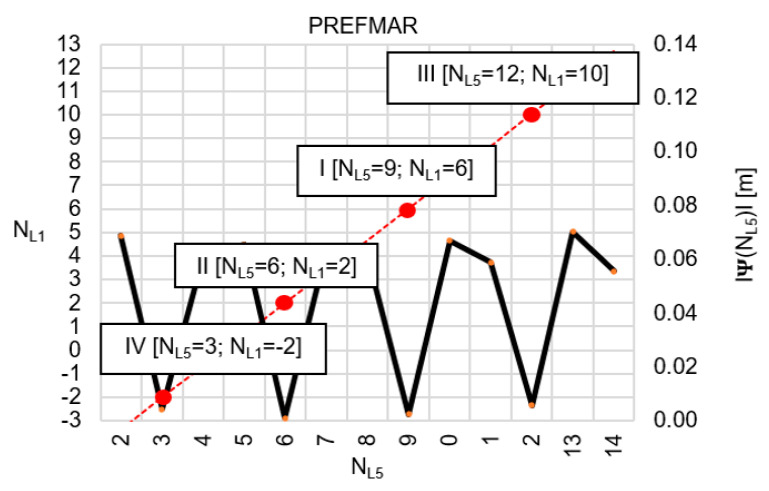
Search area of ambiguities in $N_{L5}N_{L1}$ system, for ${\tilde{N}}_{L5,0}=8.349$ and ${\tilde{N}}_{L1,0}=5.141$, with the use of the function $\left|\Psi {\left(N_{L5}\right)}_{N_{L5}N_{L1}}\right|$, for $\left|\varepsilon_{L5,L1}\right|<5.5\;\mathrm{cm}$.

Identical sets of the most probable ambiguities have been obtained based on Table 2 and Table 3, which proves the reliability of the mathematical functions $\Psi {\left(N_{L1}\right)}_{N_{L1}N_{L5}}$ and $\Psi {\left(N_{L5}\right)}_{N_{L5}N_{L1}}$ used in the PREFMAR. The lowest values of the search functions were obtained for [$N_{L1}=2;\;N_{L5}=6]$; the second proposal was [$N_{L1}=6;\;N_{L5}=9]$, and the third proposal was [$N_{L1}=10;\;N_{L5}=12]$. Similar results are obtained using the data from Table 1, for any of the individual DD epochs. The information contained in Table 2 and Table 3 can also be presented in graphical form, as shown in Figure 9 and Figure 10.

However, as already mentioned before, taking into account the accuracy of the code measurements, the most probable pair of ambiguities must be selected in a distance from a float solution below $2\lambda_{L1}$ (i.e., $\;N_{L1}^{I}=6;\;N_{L5}^{I}=9$); the search for the second pair is performed in the area $4\lambda_{L1}$ from float solution (i.e., $\;N_{L1}^{II}=2;\;N_{L5}^{II}=6$), while the third pair of ambiguities must be at a distance $6\lambda_{L1}$ (i.e., $\;N_{L1}^{III}=10;\;N_{L5}^{III}=12$). However, for the relative errors up to ±62 mm, the first four most probable pair of ambiguities will be as follows: $[N_{L1}^{I}=6;\;N_{L5}^{I}=9$], $[N_{L1}^{II}=2;\;N_{L5}^{II}=6$], $[N_{L1}^{III}=5;\;N_{L5}^{III}=8$], and $[N_{L1}^{IV}=9;\;N_{L5}^{IV}=11$].

## 8. Discussion

Generally, the search area in the PREFMAR is represented by a parallelogram. The orientation of this parallelogram in a given system of ambiguities is only dependent on the frequency of the GNSS signals transmitted. For system $N_{L1}N_{L5}$, the short sides of this parallelogram are parallel to the vertical lines of the system $N_{L1}N_{L5}$, i.e., to the $N_{L5}$ axis (Figure 9). The longer side of this parallelogram is slanted with respect to the $N_{L5}$ axis under an angle of $\alpha_{L1,L5}=tan^{-1}\left(115/154\right)=36.750655$^o^, and the geometrical center of the search area is located at point $({\tilde{N}}_{L1,0}$, ${\tilde{N}}_{L5,0}).$ Similarly, for the system $N_{L5}N_{L1}$ (Figure 10), $\alpha_{L5,L1}=tan^{-1}\left(154/115\right)=53.249344$^o^.

Please note that the presented functions fulfil the relation $\Psi {\left(N_{L1}\right)}_{N_{L1}N_{L5}}=-\Psi {\left(N_{L5}\right)}_{N_{L5}N_{L1}}$ because the values of the functions represent relative errors of phase observations between L1-L5, which can be additionally written in a simplified manner:

(36) $$\Psi {\left(N_{L1}\right)}_{N_{L1}N_{L5}}=\left[\lambda_{L5}\phi_{L5}\left(t\right)-\lambda_{L5}N_{L5}\right]-\left[\lambda_{L1}\phi_{L1}\left(t\right)-\lambda_{L1}N_{L1}\right]$$

(37) $$\Psi {\left(N_{L5}\right)}_{N_{L5}N_{L1}}=\left[\lambda_{L1}\phi_{L1}\left(t\right)-\lambda_{L1}N_{L1}\right]-\left[\lambda_{L5}\phi_{L5}\left(t\right)-\lambda_{L5}N_{L5}\right]$$

The formulas presented above also allow indicating integer ambiguities by substituting appropriate sets of ambiguities $N_{L1}$ and $N_{L5}$, near the float ambiguity, e.g., for the case presented above using 5 DD epochs, we have $\phi_{L1}\left(t\right)=$ 42.689 and $\phi_{L5}\left(t\right)=$ 36.388 thus for [$N_{L1}=6;\;N_{L5}=9]$; based on formulas (36) and (37) we obtain:

(38) $$\Psi {\left(N_{L1}\right)}_{N_{L1}N_{L5}}=\left[\lambda_{L5}*36.388-\lambda_{L5}*9\right]-\left[\lambda_{L1}*42.689-\lambda_{L1}*6\right]=-0.002\;m$$

(39) $$\Psi {\left(N_{L5}\right)}_{N_{L5}N_{L1}}=\left[\lambda_{L1}*42.689-\lambda_{L1}*6\right]-\left[\lambda_{L5}*36.388-\lambda_{L5}*9\right]=0.002\;m$$

which is equivalent to the values of the functions $\Psi {\left(N_{L1}\right)}_{N_{L1}N_{L5}}$ and $\Psi {\left(N_{L5}\right)}_{N_{L5}N_{L1}}$ obtained based on formulas (16) and (19) and presented in Table 2 and Table 3, respectively.

Presented equations of ambiguity functions can be used for different double or triple frequencies; however, they have specific properties in every case. Therefore, we are going to present the most popular combinations of GNSS frequencies in the near future to show the power of the PREFMAR.

It should be emphasized that the PREFMAR gives results based on GNSS measurements from only two available GNSS satellites (see Section 7); therefore, we can use this method effectively in challenging observational satellite conditions.

Furthermore, to obtain the most accurate float solutions, we can effectively use the Kalman filter in differential [28] or relative code GNSS positioning.

## 9. Summary and Conclusions

This research presents a precise and fast method of ambiguity resolution (PREFMAR) for indicating the most probable ambiguities for GNSS observations transmitted on two frequencies, i.e., L1/E1 = 1575.42 MHz and L5/E5a = 1176.45 MHz. Signals with such frequencies are transmitted by satellites of the American GPS system and the European GALILEO system. Therefore, the combination of these frequencies allows the precise integration of the various GNSS positioning systems. The described method is used to determine ambiguities using only one observation epoch immediately. This PREFMAR is intended for both short and long baselines. Its efficiency mostly depends on the values of relative errors in DD phase observations and on the precision of code measurements. Ambiguity is chosen based on mathematical functions $\Psi {\left(N_{L1}\right)}_{N_{L1}N_{L5}}$ or $\Psi {\left(N_{L5}\right)}_{N_{L5}N_{L1}}$, using a correlation between the value of a precisely defined ambiguity and dependent only on the frequency of satellite signals. The PREFMAR allows the ambiguities for single measurement epochs to be determined without using the VC matrix from the float solution and also allows the most probable ambiguities to be indicated even for individual DD observations. The presented mathematical functions can also be used for precise and immediate re-initialization of the ambiguities. Additionally, the contribution presents interpretations of the derived functions $\Psi {\left(N_{L1}\right)}_{N_{L1}N_{L5}}$ and $\Psi {\left(N_{L5}\right)}_{N_{L5}N_{L1}}$ in relation to the relative errors in phase observations. As a result, a simplified calculation method is included for values of the functions $\Psi {\left(N_{L1}\right)}_{N_{L1}N_{L5}}\;\mathrm{and}\;\Psi {\left(N_{L5}\right)}_{N_{L5}N_{L1}}$, which are equivalent to the mathematically detailed versions, but only in the range $0.5\lambda_{L5}$ for function $\Psi {\left(N_{L1}\right)}_{N_{L1}N_{L5}}$ and in the range $0.5\lambda_{L1}$ for function $\Psi {\left(N_{L5}\right)}_{N_{L5}N_{L1}}$.

## Figures and Tables

**Table 1:** DD observations for the G01–G06 satellite pair.

Epoch	C1 (m)	L1 (c)	P5 (m)	L5 (c)	N_L1_ (c)	N_L5_ (c)
1	7.437	42.686	6.967	36.390	6.074	9.050
2	7.093	42.725	7.073	36.407	5.556	8.651
3	5.402	42.654	7.132	36.365	5.175	8.378
4	7.393	42.699	7.333	36.394	4.164	7.618
5	3.771	42.681	7.221	36.384	4.734	8.047
Average	6.219	42.689	7.145	36.388	5.141	8.349

**Table 2:** The set of integer candidates based on the function $\Psi {\left(N_{L1}\right)}_{N_{L1}N_{L2}}$, for ${\tilde{N}}_{L1,0}=5.141$ and ${\tilde{N}}_{L5,0}=8.349$, where $N_{L1,i}\in <-3;13>$.

$N_{L1}$	${\tilde{N}}_{L5}$	$\Psi {\left(N_{L1}\right)}_{N_{L1}N_{L5}}\;(m)$	Solution No.	$N_{L5}={\left[{\tilde{N}}_{L5}\right]}_{roundoff}$
−3	2.270	0.069		2
−2	3.016	0.004	IV	3
−1	3.763	−0.060		4
0	4.510	−0.125		5
1	5.257	0.065		5
2	6.003	0.001	II	6
3	6.750	−0.064		7
4	7.497	0.127		7
5	8.244	0.062		8
6	8.990	−0.002	I	9
7	9.737	−0.067		10
8	10.484	0.123		10
9	11.231	0.059		11
10	11.977	−0.006	III	12
11	12.724	−0.070		13
12	13.471	0.120		13
13	14.218	0.055		14

**Table 3:** Set of integer candidates based on the function $\Psi {\left(N_{L5}\right)}_{N_{L5}N_{L1}}$, for ${\tilde{N}}_{L5,0}=8.349$ and ${\tilde{N}}_{L1,0}=5.141$, where $N_{L5}\in <2;14>$.

$N_{L5}$	${\tilde{N}}_{L1}$	$\Psi {\left(N_{L5}\right)}_{N_{L5}N_{L1}}\;(m)$	Solution No.	$N_{L1}={\left[{\tilde{N}}_{L1}\right]}_{roundoff}$
2	−3.361	−0.069		−3
3	−2.022	−0.004	IV	−2
4	−0.683	0.060		−1
5	0.656	−0.065		1
6	1.995	−0.001	II	2
7	3.335	0.064		3
8	4.674	−0.062		5
9	6.013	0.002	I	6
10	7.352	0.067		7
11	8.691	−0.059		9
12	10.030	0.006	III	10
13	11.369	0.070		11
14	12.708	−0.055		13
